# Managing ocular allergy in resource-poor settings

**Published:** 2017-02-10

**Authors:** Millicent Bore

**Affiliations:** 1 Lecturer: Department of Ophthalmology, College of Health Sciences, University of Nairobi, Kenya. **millicentbore@gmail.com**

Ocular allergy is a common inflammatory condition seen almost daily at the outpatient clinic. It occurs because the ocular surface is exposed to a variety of allergens, making it susceptible to allergic reactions. The hallmark of the disease is **itching,** and the clinical symptoms and signs are **bilateral** and vary according to individual cases.

The common predisposing factors of ocular allergy include environmental allergens, genetic predisposition to atopic reactions and hot, dry environments.

The patient may have associated systemic features like eczema, asthma and rhinitis.

## Types of ocular allergy

Ocular allergies can be divided into:

1. Vernal keratoconjunctivitis
2. Atopic keratoconjunctivitis
3. Acute allergic conjunctivitis (includes seasonal and perennial allergic conjunctivitis)
4. Giant papillary conjunctivitis

The first two forms of ocular allergies are sight-threatening. Both can lead to damage of the cornea by causing ulcers and scarring (secondary to inflammation of the ocular surface), ultimately leading to vision loss.

### Vernal keratoconjunctivitis

**Figure 1.**
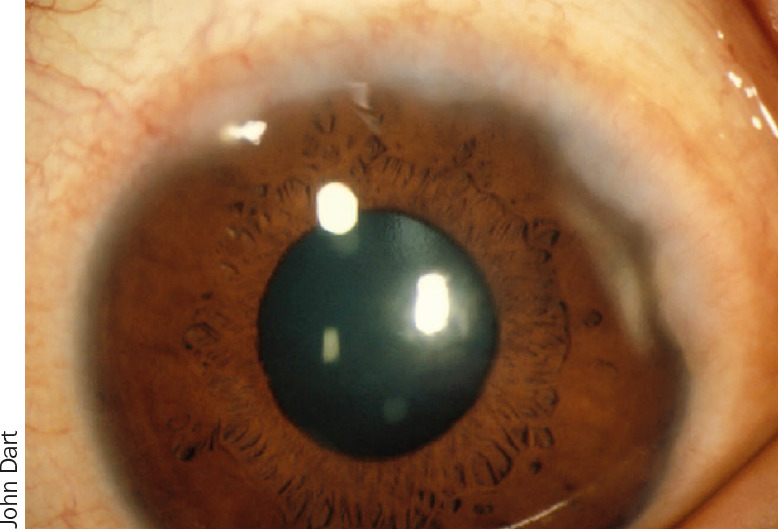
Vernal keratoconjunctivitis showing Injection and swelling at the limbus with conjunctivalisation of the cornea

Onset of vernal keratoconjunctivitis is usually in childhood (mean age 7 years) and it tends to become less severe by the late teens. It is more common in boys than in girls. If left untreated, it can result in corneal conjunctivalisation and scarring (Figure 1). The symptoms are severe itching, watering, foreign body sensation and thick mucus discharge.

**Figure 2.**
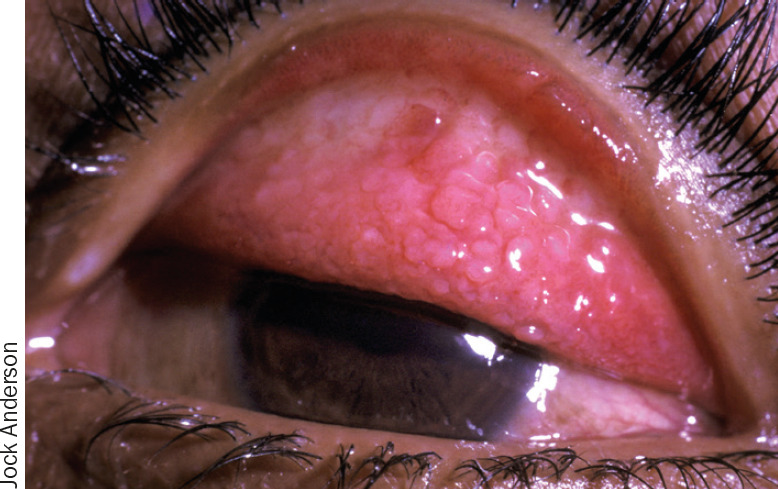
Papillae on the everted upper eyelid in vernal keraconjunctivitis

**Figure 3.**
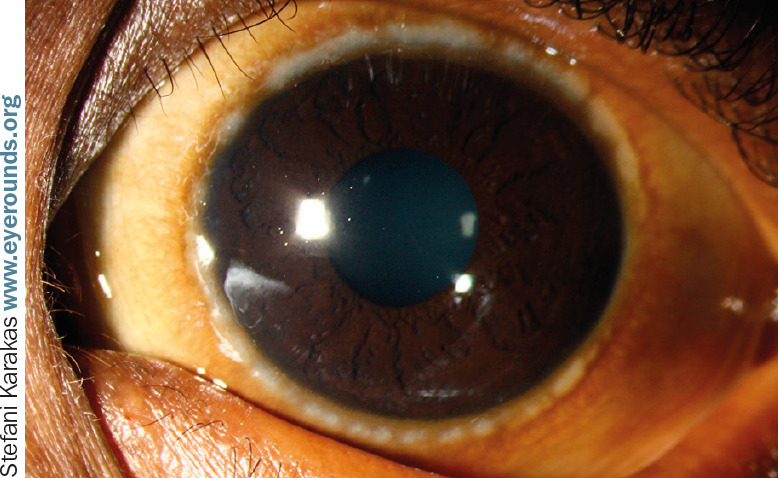
Horner-Trantas dots in a child with vernal conjunctivitis

**Signs:** The hallmark sign of vernal keratoconjunctivitis is papillae formation in the tarsal conjunctiva; these can be large and irregular (known as cobblestone papillae) (Figure 2). There is conjunctival injection and/or hyperpigmentation and there may be peri-limbal small white dots (Horner-Trantas dots) (Figure 3). The limbus can become pigmented and the cornea can be affected with plaques and ulceration of the upper cornea.

**Figure 4.**
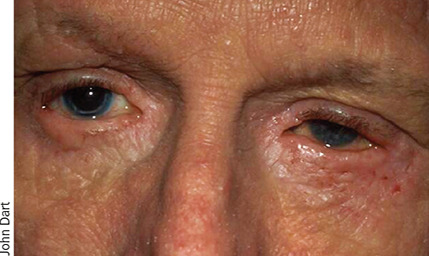
Atopic keratoconjunctivitis

### Atopic keratoconjunctivitis

Atopic keratoconjunctivitis classically presents in adulthood and has a chronic and unremitting course.

**History:** History of atopy (asthma, eczema). Severe itching, watering, foreign body sensation, mucus discharge. Symptoms occur year-round.

**Signs:** Skin changes on the eyelids, e.g. erythema, dryness, scaliness and thickening. Papillae on the tarsal conjunctiva. In severe cases, conjunctival scarring and forniceal shortening may be present.

**Other ocular allergies**

These include acute allergic conjunctivitis (seasonal and perennial allergic conjunctivitis) and giant papillary conjunctivitis. Predisposing factors for giant papillary conjunctivitis include contact lens wear and irritation from exposed sutures or a prosthesis.

**NOTE:** All ocular allergies can have sight-threatening complications if not managed well, e.g. keratoconus (due to excessive rubbing) and glaucoma (due to the prolonged use or misuse of steroids).

**How do ocular allergies develop?**

**Figure 5:**
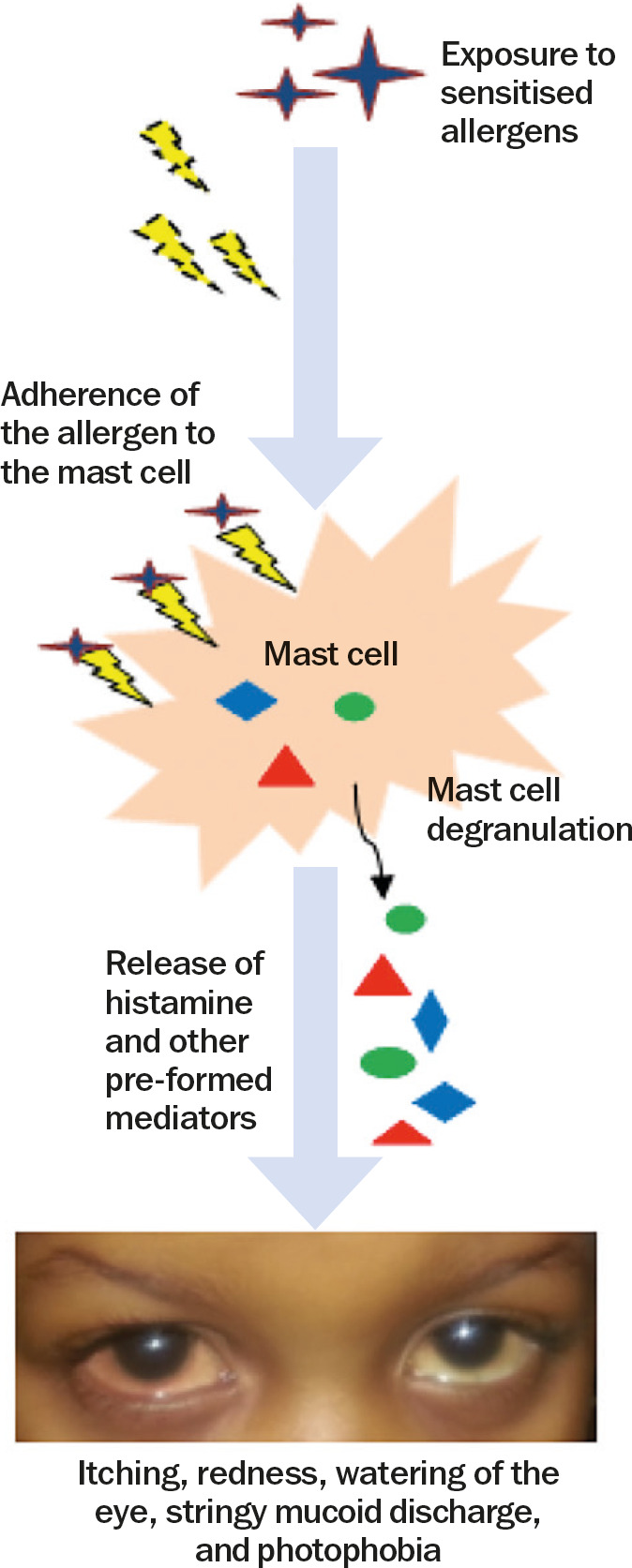
The ocular allergy cascade in a sensitised individual

The basic mechanism of these conditions is type-1 hypersensitivity. The inflammatory response in vernal and atopic keraconjunctivitis is due to inflammatory mediators, mainly from mast cells (Figure 5).

## Grading of clinical severity

There is no globally accepted system or guidelines for the grading and management of ocular allergy, although several authors have proposed such systems.^1–5^

All patients with ocular allergy should be graded according to the level of severity.^6^ This is because the grade of severity has an impact on clinical decision making and helps ascertain the patients’ ocular clinical status and risk of vision loss. It also helps to determine the choice of treatment and the timing/frequency of follow-up.

Table 1 is based on a simplified clinical grading system which the authors have developed for use in Kenya and which applies to all ocular allergies. It takes into consideration the clinical signs present during the objective assessment but not the patient's symptoms.

## Treatment

The management of ocular allergies in low- and middle-income countries is complicated by the high cost of drugs and the limited options available

Table 2 details the treatment guidelines developed for use in Kenya, based on the severity grading.

**Note:** Patients diagnosed with vernal or atopical keratoconjunctivitis should always be treated as **‘severe’** cases, whatever their presenting clinical signs.

There are many tools that can be used in the management of ocular allergy.

**Non-pharmacological treatment,** including allergen avoidance and cold compresses, are important for providing short-term relief from symptoms. The patient should also be advised to avoid eye rubbing.

**Topical lubricants,** preferably preservative free, are recommended for use in all grades of severity to dilute allergens and reverse tear film instability secondary to chronic inflammation.

**Table 1.** A grading guide based on the Ocular Allergy Clinical Grading Guide developed for use in Kenya. The grading is determined by the most severe sign present in the most severely affected eye

Grade 1	Mild	Moderate	Severe
**Papillae**	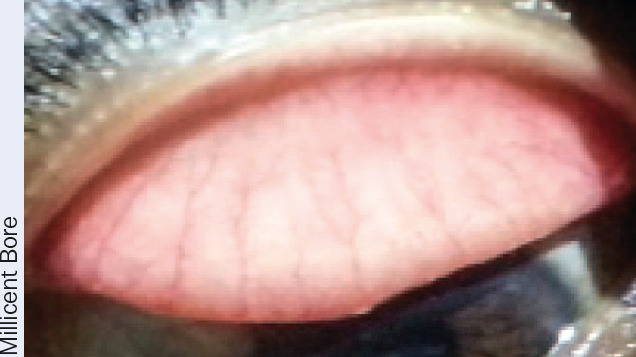 Micro: <0.3mm	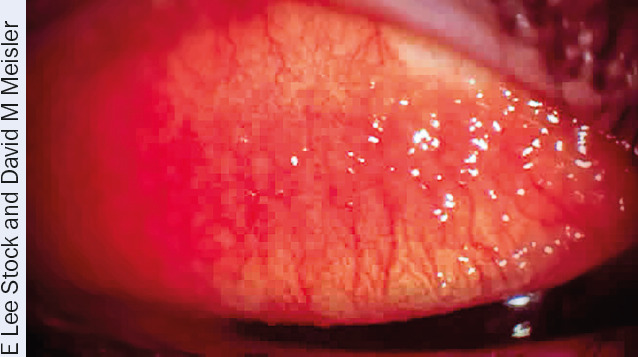 • Macro: between 0.3 and 0.5 mm • +/− Fibrosis	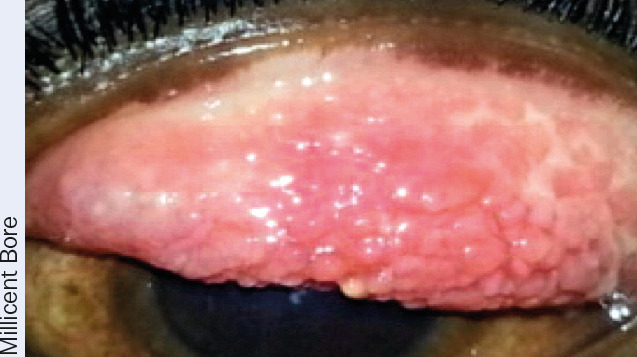 • Cobblestone papillae: > 0.5mm but smaller than 1.0 mm • Giant papillae: >1.0 mm
**Conjunctiva**	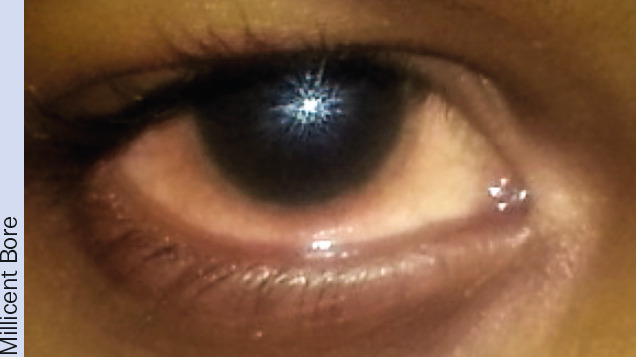 Hyperemia	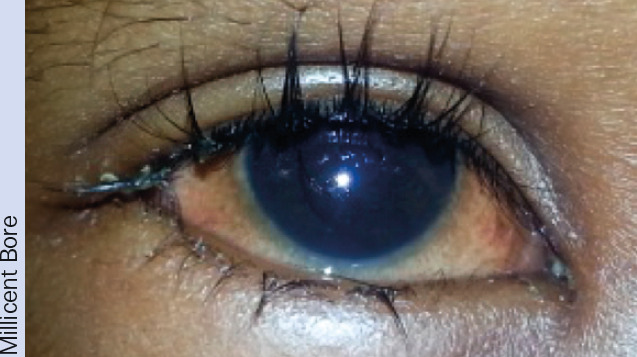 • Hyperemia • Diffuse thin chemosis	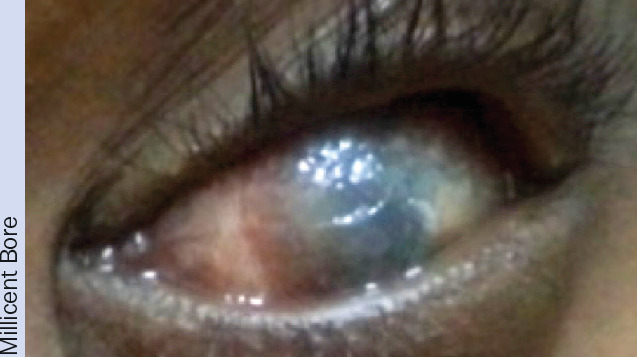 • Hyperemia • Cyst-like chemosis/scar • Conjunctivalisation of the cornea
**Limbus (limbal oedema or Horner-Trantas dots)**	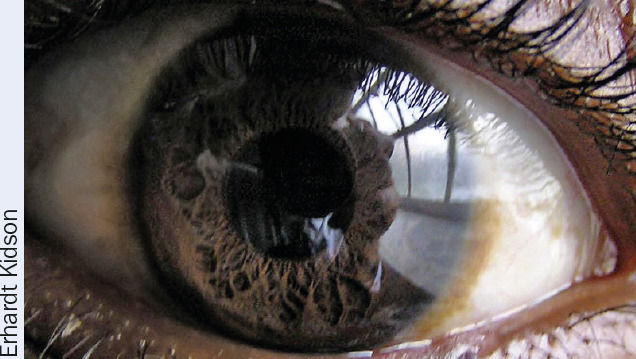 No manifestations	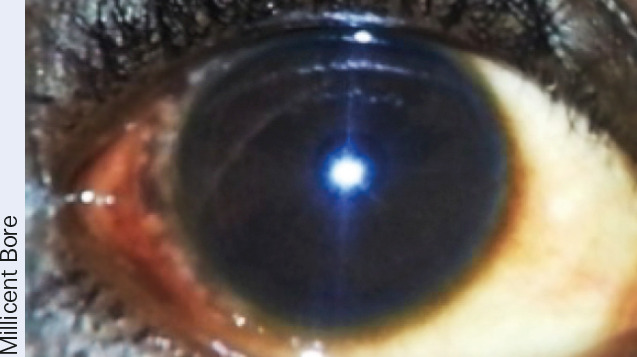 < ½ of limbal circumference affected	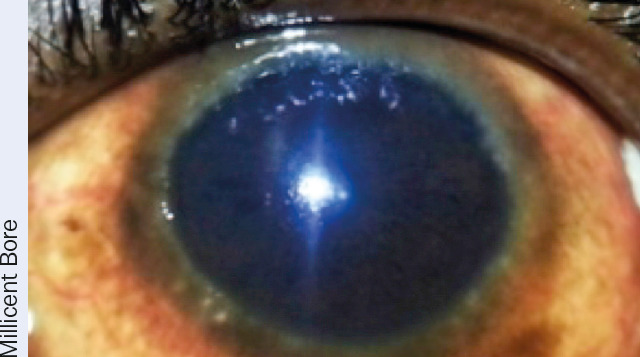 ½ or more of limbal circumference affected
**Cornea**	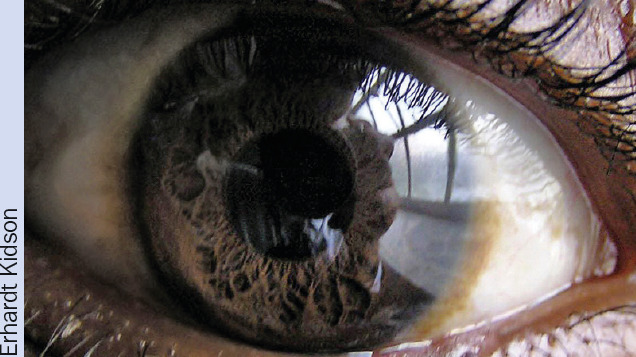 Clear	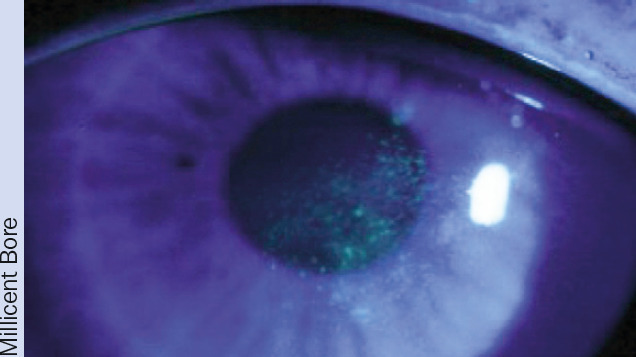 Superficial punctate keratitis	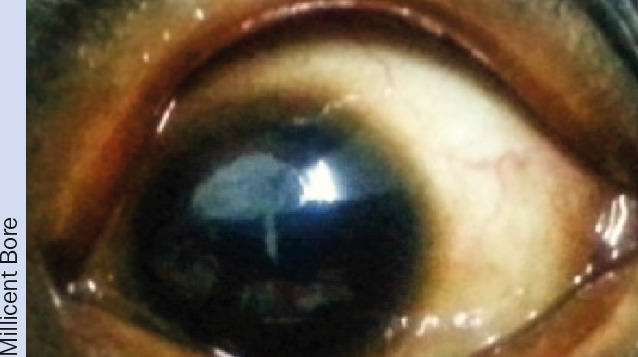 • Shield ulcer/epithelial erosion • Keratoconus +/− central leucoma

Note that patients diagnosed with vernal or atopic keraconjunctivitis should be treated as **‘severe’** cases, whatever their presenting clinical signs.

**Table 2.** Treatment and follow-up guidelines, based on severity grading (developed for Kenya)

Grade	Mild	Moderate	Severe
**Treatment**	Topical antihistamine (e.g. Emedastine) for 1 month **OR** Multi-action drug, e.g. olopatadine, for 1 month	Mild topical steroid, e.g. fluoromethalone 4 times a day for 1–2 weeks +/− steroid ointment at night for 2–4 weeks Mast-cell stabiliser (e.g. cromolyn sodium)	Pulsed topical steroid regimen (start frequently then taper) +/− topical cyclosporine 0.5–2% until good remission, then stop. Topical antihistamine + mast cell stabiliser/ multi-action drug for 1 month then mast cell stabiliser for maintenance Steroid ointment at night for 2–4 weeks Cobblestone/giant papillae or refractory cases: subtarsal steroid* (e.g. triamcinolone) Shield ulcer: corneal scraping/superficial keratectomy + topical steroid-antibiotic +/− mydriatic
**Follow-up**	As required	Review after 4–6 weeks, then – if stable – as required	Review after 1–2 weeks then monthly while on steroids Taper steroids (check IOP) Stagger reviews to 3-monthly once patient is stable

* Avoid repeated use or use in children aged less than 10 years due to the risk of elevated IOP

**Topical antihistamines and mast cell stabilisers** are considered as first-line treatment. Mast cell stabilisers require a loading period of up to two weeks in order to achieve maximal efficacy. It should be combined with an antihistamine (short duration of action) or a mild topical steroid such as fluoromethalone to provide faster relief. Mast cell therapy should be continued when the steroids are stopped.

**Dual-action drugs** have both antihistamine and mast cell stabiliser action. They are effective in treating ocular allergy and outperform other groups of drugs. Another benefit is improved compliance because of a reduction in the number of medications to be used.

**Topical ocular steroids** are effective (probably the most effective of all options), but pose the important risk of frequent side effects (glaucoma, cataracts, corneal ulcers). Mild topical steroids should be used in acute crises for short periods of time; preferably less than 2 weeks. In cases of severe ocular allergy, a pulsed topical steroid regimen (start frequently, then taper) is advised. The duration of use is based on the grade of severity. Steroid ointments can be used at night for a short duration.

The use of **supra-tarsal steroids** is recommended only for severe cases where topical medication does not control symptoms or when there is disease progression (refractory cases). Their use is also recommended in patients with severe papillary reaction leading to corneal epithelial erosions/shield ulcers.^6^

**‘All patients and their carers should be counselled.’**

**Topical immunomodulators,** such as cyclosporin A, have been shown to be of great benefit as steroid-sparing agents in chronic disease^7^, although they are not readily available.

## Patient counselling

All patients and their carers should be counselled. A well-informed patient and parent/guardian will be in a better position to take part in the management of the condition. Counselling leads to improved compliance with medication and follow-up visits. It also leads to a reduction in self-medication, which in turn reduces possible misuse of steroids. It is important to make patients with sight-threatening disease aware that it can be blinding, so that they can understand the importance of proper follow-up and keeping their appointments.

Counselling can also help patients to avoid the complications associated with chronic eye rubbing (keratoconus) and the overuse or misuse of steroids (glaucoma, cataract, etc.).

Talk to patients about what they can do to support themselves, e.g. avoiding allergens, using cool compresses and preservative-free artificial tears, and wearing spectacles or sunglasses when outside. Basic printed information can be issued to patients during clinic visits.

## Follow-up

Frequency of follow-up is linked to:

- Clinical severity grading
- Sight-threatening or non sight-threatening condition?
- Clinical response to treatment

A follow-up visit should include recent history, measurement of visual acuity, and slit lamp biomicroscopy. If corticosteroids are prescribed, measurement of intraocular pressure and pupillary dilation should be performed to evaluate for glaucoma and cataract.

If there is inadequate correction of refractive error and a history of frequent changes in spectacle prescriptions, suspect keratoconus. Look out for infections such as viral keratitis and refer all patients with severe disease (i.e. those developing complications) or those not responding to treatment.
